# Socioeconomic status and risk of COVID-19 hospitalization in the *All of Us* Research Program

**DOI:** 10.3389/fpubh.2025.1690430

**Published:** 2025-12-10

**Authors:** Angela Choi, Andrew Craver, Jiajun Luo, Yuqing Yang, Briseis Aschebrook-Kilfoy

**Affiliations:** 1 Department of Earth, Environmental, and Planetary Sciences, Northwestern University, Evanston, IL, United States; 2 Institute for Population and Precision Health, University of Chicago, Chicago, IL, United States; 3 Department of Surgery, The University of Chicago, Chicago, IL, United States; 4 Department of Family Medicine, The University of Chicago, Chicago, IL, United States

**Keywords:** COVID-19, socioeconomic status, deprivation index, *All of Us* Research Program, socioeconomic disparities, COVID-19 vaccination, United States

## Abstract

**Introduction:**

The relationship between socioeconomic status and COVID-19 related hospitalization has been widely examined, although findings differ across study settings and populations. This study used data from the NIH *All of Us* Research Program to explore the association between age, race, income, education, neighborhood deprivation, and risk of COVID-19 hospitalization while controlling for key covariates, including risk factors and vaccination status.

**Methods:**

This cross-sectional analysis included 25,650 adults with confirmed COVID-19 between 2021 and 2023, including 662 hospitalized for COVID-19. Covariates were age, sex at birth, race/ethnicity, annual household income, education, and deprivation index score. Our analysis controlled for the following risk factors: BMI, smoking status at enrollment, COVID-19 vaccination status, and history of cancer, chronic kidney disease, chronic obstructive pulmonary disease, diabetes, and hypertension. Logistic regression was used to estimate odds ratios.

**Results:**

Increased hospitalization risk was observed among participants aged 66–95 (OR: 1.81, 95% CI: 1.27, 2.58) and aged 51–65 (OR: 1.73, 95% CI: 1.24, 2.43), as well as Non-Hispanic Black/African American participants (OR: 2.48, 95% CI: 1.99, 3.10), and Hispanic participants (OR: 1.47, 95% CI: 1.15, 1.88). Increased risk was also observed among participants living in the highest deprivation areas (OR: 2.60, 95% CI: 2.04, 3.31), those with an annual income less than $25,000 (OR: 1.67, 95% CI: 1.15, 2.44), and those with an annual income of $25,000 to $50,000 (OR: 1.45, 95% CI: 1.06, 1.99).

**Conclusion:**

Our findings indicate meaningful associations between the risk of COVID-19-associated hospitalization and socioeconomic factors including age, racial/ethnic minority status, lower income, and higher area deprivation.

## Introduction

The full number of COVID-19 hospitalizations in the US remains unknown, with estimates as high as 6 million (1). As COVID-19 remains an important public health concern, understanding how hospitalization rates have differed historically across demographic and socioeconomic groups can inform policy and interventions.

Age is a strong predictor of COVID-19 hospitalization risk, particularly among those 65 and older (1). Race and ethnicity are also linked to differences in hospitalization rates; meta-analyses of studies on COVID-19 outcomes report excess risk of hospitalization among Black/African Americans and Hispanic Americans (2–4), with evidence of increased risk among Asian Americans as well (4).

Prior research has shown that socioeconomic factors, especially income and neighborhood-level deprivation, further contribute to disparities in COVID-19 outcomes (4). Studies using neighborhood-level indices such as the Social Vulnerability Index (SVI) and Area Deprivation Index (ADI) have found that residents of highly deprived areas experienced greater infection, hospitalization, and mortality rates (5–7). Many of these studies were ecological in design, relying on county- or state-level data, and were conducted during the early, pre-vaccine phase of the pandemic (8, 9).

This study advances previous research by using participant-level data from the National Institutes of Health (NIH) *All of Us* Research Program, a large and demographically diverse U.S. cohort that links survey-derived socioeconomic status data with electronic health-record (EHR) data across multiple states. While earlier research has examined associations between socioeconomic status (SES) and COVID-19 hospitalization, most focused on a limited set of SES indicators or region-specific populations. Few have assessed a broader set of individual-level factors including age, race and ethnicity, income, education, and neighborhood deprivation within a single national dataset. Using data from 25,650 confirmed COVID-19 cases between 2021 and 2023, this study offers a nationwide analysis spanning multiple pandemic years, evaluating how demographic, socioeconomic, and clinical factors—together with comorbidities and vaccination status—shape hospitalization risk at the individual level.

## Methods

This work was performed on data from the NIH *All of Us* Research Program, a diverse nationwide cohort of over 800,000. Data were accessed via the *All of Us* Researcher Workbench, the program’s online data repository and research environment. Despite temporary pauses in in-person enrollment and data collection during the pandemic, EHR data for the cohort continued to be collected, allowing for capture of hospitalization trends throughout the study period (10).

We first created a research cohort consisting of participants whose EHR was linked to the rest of their *All of Us* data. This cohort was further refined to include only participants who self-identified as non-Hispanic White, non-Hispanic Black, Hispanic, or Asian, yielding a cohort of 308,741.

COVID-19 cases included all entries in the *Conditions* domain of the Workbench dataset builder (SNOMED code 840539006), as well as positive COVID-19 test results from the *Labs and Measurements* domain (LOINC 94500–6). Cases were further coded as *inpatient* if the entry was categorized as an inpatient encounter in the *Visit Occurrence Concept Name* column and as a primary encounter in the *Condition Type Concept Name* column of the query. COVID-19 cases and hospitalizations were restricted to those that occurred from 2021 to 2023. This restriction ensured that vaccination status could be accurately incorporated as a covariate, so that hospitalization risk reflected both preexisting comorbidities and vaccine-era infection dynamics.

COVID-19 risk factors (28) were also ascertained from the *Conditions* domain history of the Workbench dataset builder. These covariates were: history of any cancer (SNOMED code 3633460000), chronic kidney disease (CKD) (SNOMED code 709044004), chronic obstructive pulmonary disease (COPD) (SNOMED code 13645005), type I and II diabetes mellitus (SNOMED code 73211009), and hypertension (SNOMED code 38341003). Participants whose earliest EHR entry for any of these conditions occurred after 2020 were excluded from the dataset.

COVID-19 vaccination status (RXNORM 2468231) was ascertained from the *Drugs* domain of the Workbench dataset builder, as well as from *COVID-19 Vaccine Survey* responses. Participants were coded as having received the COVID-19 vaccine if they had either an EHR-recorded record of receiving the vaccine, or if they responded *yes* to the survey question “Did you receive the first dose of the COVID-19 vaccination?”

Additional covariates were ascertained from other *All of Us* data sources. Participant age, race/ethnicity, sex assigned at birth, and BMI were collected at recruitment. Educational attainment and annual household income were ascertained from the *Basics* survey, while current smoking status was ascertained from the *Lifestyle* survey.

Deprivation index score, available in the Workbench *Zip Code Socioeconomic Status* domain, is a composite score generated by the *All of Us* Data and Research Center using variables from the 2017 American Community Survey (ACS) and assigned to the 3-digit zip code prefix level. The ACS items included in the calculation of the deprivation index score were the fraction of households receiving public assistance income or food stamps or SNAP in the past 12 months, the fraction of the population aged 25 and older with educational attainment of at least high school graduation, the median household income in the past 12 months in 2015 inflation-adjusted dollars, the fraction of the population with no health insurance coverage, the fraction of the population with income in past 12 months below the poverty level, and the fraction of vacant houses in the 3-digit zip code prefix area. A deprivation index score closer to 1 indicates a higher degree of socioeconomic deprivation. Deprivation index score was binned into natural quartiles using the Python qcut function.

The final dataset included 25,650 COVID-19 cases and 662 COVID-19 hospitalizations: 325 in 2021, 261 in 2022, and 76 in 2023.

Multiple imputation was performed to fill missing variables using the R package Multivariate Imputation by Chained Equations (MICE). Logistic regression was used to estimate the odds ratio and 95% confidence interval for hospitalization. Data cleaning was performed using Python version 3.10.12 and analysis was performed using R version 4.4.0.

## Results

Table 1 presents an overview of our sample. A total of 662 participants in our dataset had at least one EHR-documented inpatient hospitalization for COVID-19. Most (75.1%) of these participants were 51–95 years old, 36.3% had an annual household income of less than $25,000, and 42.9% had a high school education or had not completed high school. Non-Hispanic White participants accounted for 35.8% of inpatient cases, while 40.2% were among non-Hispanic Black/African American participants, and 22.4% were among Hispanic participants. There were fewer than 20 cases among Asian participants. Forty-eight percent of participants lived in areas that fell within the highest deprivation index score quartile, while 15.6% lived in areas that fell within the lowest deprivation index score quartile.

**Table 1:** Study population overview.

Characteristic		*All of Us* Participants with Linked EHR		*All of Us* Participants with COVID-19		*All of Us* Participants with COVID-19 Hospitalization	
		**N**	**%**	**N**	**%**	**N**	**%**
**Total**		308,741	100%	25,650	100%	662	100%
**Age**	19-35	54,971	17.80%	3,381	13.18%	49	7.40%
	36-50	69,599	22.54%	5,630	21.95%	116	17.52%
	51-65	89,145	28.87%	7,592	29.60%	243	36.71%
	66-95	95,026	30.78%	9,047	35.27%	254	38.37%
**Sex at Birth**	Female	190,500	61.70%	16,993	66.25%	403	60.88%
	Male	115,469	37.40%	8,470	33.02%	250	37.76%
	Other Sex	289	0.09%	~	~	~	~
	Missing	2,483	0.80%	168	0.65%	~	~
**Race/Ethnicity**	NH White	178,267	57.74%	15,942	62.15%	237	35.80%
	NH Black/AA	58,019	18.79%	3,979	15.51%	266	40.18%
	Hispanic	62,141	20.13%	5,031	19.61%	148	22.36%
	Asian	10,314	3.34%	698	2.72%	~	~
**Income**	< $25k	73,092	23.67%	5,183	20.21%	240	36.25%
	$25k - $50k	46,309	15.00%	4,028	15.70%	92	13.90%
	$50k - $100k	60,134	19.48%	5,451	21.25%	69	10.42%
	> $100k	70,342	22.78%	6,264	24.42%	67	10.12%
	Missing	58,864	19.07%	4,724	18.42%	194	29.31%
**Education**	Finished College	138,759	44.94%	11,836	46.14%	194	29.31%
	Some College	78,928	25.56%	7,096	27.66%	160	24.17%
	High School	57,596	18.66%	4,377	17.06%	180	27.19%
	< High School	27,158	8.80%	1,914	7.46%	104	15.71%
	Missing	6,300	2.04%	427	1.66%	24	3.63%
**Deprivation Index Score**	Q1 (0.149 - 0.283)	77,050	24.96%	7,452	29.05%	103	15.56%
	Q2 (0.283 - 0.314)	76,382	24.74%	6,562	25.58%	130	19.64%
	Q3 (0.314 - 0.361)	75,538	24.47%	5,828	22.72%	105	15.86%
	Q4 (0.361 - 0.626)	76,746	24.86%	5,575	21.73%	318	48.04%
	Missing	3,025	0.98%	233	0.91%	~	~
**BMI**	Normal	78,014	25.27%	5,362	20.90%	134	20.24%
	Underweight	4,168	1.35%	283	1.10%	~	~
	Overweight	89,086	28.85%	7,058	27.52%	178	26.89%
	Obese	117,608	38.09%	11,715	45.67%	331	50.00%
	Missing	19,865	6.43%	1,232	4.80%	~	~
**Smoking Status**	No	252,127	81.66%	22,447	87.51%	493	74.47%
	Yes	46,297	15.00%	2,452	9.56%	142	21.45%
	Missing	10,317	3.34%	751	2.93%	27	4.08%
**Cancer**	Yes	42,862	13.88%	5,558	21.67%	187	28.25%
	No	265,879	86.12%	20,092	78.33%	475	71.75%
**CKD**	Yes	21,446	6.95%	3,509	13.68%	198	29.91%
	No	287,295	93.05%	22,141	86.32%	464	70.09%
**COPD**	Yes	19,050	6.17%	2,861	11.15%	175	26.44%
	No	289,691	93.83%	22,789	88.85%	487	73.56%
**COVID-19 Vaccination Status**	Yes	110,619	35.83%	11,888	46.35%	240	36.25%
	No	198,122	64.17%	13,762	53.65%	422	63.75%
**Diabetes**	Yes	50,379	16.32%	7,089	27.64%	300	45.32%
	No	258,362	83.68%	18,561	72.36%	362	54.68%
**Hypertension**	Yes	112,118	36.31%	14,280	55.67%	467	70.54%
	No	196,623	63.69%	11,370	44.33%	195	29.46%

~ In accordance with *All of Us* guidelines, results are not presented for an *n* of fewer than 20 participants.

Table 2 presents the results of multivariable logistic regression. Compared to participants aged 19–35, increased risk of hospitalization was observed among those aged 51–65 (OR: 1.73, 95% CI: 1.24, 2.43), and 66–95 (OR: 1.81, 95% CI: 1.27, 2.58).

**Table 2:** Multivariable logistic regression.

**Covariate**		**OR (95% CI)**	**P-Value**	**Sig.**
**Age**	19-35	Ref		
	36-50	1.34 (0.95, 1.89)	0.101	-
	51-65	1.73 (1.24, 2.43)	0.001	***
	66-95	1.81 (1.27, 2.58)	0.001	***
**Sex at Birth**	Female	Ref		
	Male	1.14 (0.96, 1.35)	0.149	-
	Other Sex	~	~	-
**Race/Ethnicity**	NH White	Ref		
	Asian	~	~	-
	Hispanic	1.47 (1.15, 1.88)	0.003	**
	NH Black	2.48 (1.99, 3.10)	0.000	***
**Income**	>$100K	Ref		
	<$25k	1.67 (1.15, 2.44)	0.019	*
	$25k - $50k	1.45 (1.06, 1.99)	0.022	*
	$50k - $100k	1.03 (0.75, 1.41)	0.853	-
**Education**	Finished College	Ref		
	Some College	0.92 (0.76, 1.11)	0.128	-
	High School	1.19 (0.96, 1.46)	0.281	-
	< High School	1.21 (0.93, 1.56)	0.398	-
**Deprivation Index Score**	Q1	Ref		
	Q2	1.09 (0.83, 1.44)	0.518	-
	Q3	0.92 (0.69, 1.23)	0.580	-
	Q4	2.60 (2.04, 3.31)	0.000	***
**COVID-19 Vaccination**	Yes	Ref		
	No	1.28 (1.08, 1.52)	0.005	**
**BMI**	Normal	Ref		
	Underweight	~	~	-
	Overweight	0.93 (0.74, 1.18)	0.558	-
	Obese	0.88 (0.71, 1.10)	0.271	-
**Current Smoker**	No	Ref		
	Yes	1.77 (1.41, 2.22)	0.000	***
**Cancer**	No	Ref		
	Yes	1.33 (1.10, 1.61)	0.003	**
**CKD**	No	Ref		
	Yes	1.64 (1.34, 2.01)	0.000	***
**COPD**	No	Ref		
	Yes	1.58 (1.28, 1.94)	0.000	***
**Diabetes**	No	Ref		
	Yes	1.25 (1.04, 1.51)	0.020	*
**Hypertension**	No	Ref		
	Yes	0.94 (0.76, 1.16)	0.547	-

~ In accordance with *All of Us* guidelines, results are not presented for an *n* of fewer than 20 participants.

Significance codes: *** 0.001, ** 0.01, * 0.05.

Compared to non-Hispanic White participants, increased risk was observed among non-Hispanic Black/African American participants (OR: 2.48, 95% CI: 1.99, 3.10) and Hispanic participants (OR: 1.47, 95% CI: 1.15, 1.88).

Compared to participants with an annual household income over $100,000, increased risk was observed among those with an income of less than $25,000 (OR: 1.67, 95% CI: 1.15, 1.88) and those with an income of $25,000 to $50,000 (OR: 1.45, 95% CI: 1.06, 1.99).

Participants living in areas that fell within the highest deprivation index quartile, with a score of 0.361–0.626, demonstrated increased risk of hospitalization (OR: 2.60, 95% CI: 2.04, 3.31), compared to those living in the lowest deprivation index quartile (0.149–0.283).

Smoking (OR: 1.77, 95% CI: 1.41, 2.22), history of cancer (OR: 1.33, 95% CI: 1.10, 1.61), history of chronic kidney disease (OR: 1.64, 95% CI: 1.34, 2.01), history of chronic obstructive pulmonary disease (OR: 1.58, 95% CI: 1.28, 1.94), and history of diabetes (OR: 1.25, 95% CI: 1.04, 1.51) were all associated with increased risk of hospitalization. Participants with no history of COVID-19 vaccination were more likely to be hospitalized (OR: 1.28, 95% CI: 1.08, 1.52).

## Discussion

Our analysis showed that age, race, income, and neighborhood-level deprivation had strong independent associations with COVID-19 hospitalization. These relationships align with prior studies identifying social and structural determinants of hospitalization risk, although regional analyses have reported variation as to which factors are significant. For example, in a 12-state cohort of more than 11,000 hospitalized patients, researchers found differences by race in inpatient mortality after adjusting for age, sex, insurance status, comorbidities, neighborhood deprivation, and site of care (11). In North and South Carolina, a retrospective analysis of approximately 13,000 adults found higher odds of inpatient hospitalization among Black/African American and Hispanic patients; however key limitations of this study included lack of adjustment for age, sex, comorbidities, and socioeconomic variables within each racial and ethnic group (12). Our adjusted nationwide model accounts for these covariates and confirms the same pattern at a broader scale, extending the regional findings to a diverse U.S. cohort.

Structural and occupational exposures may account for the higher odds of hospitalization among Black/African American and Hispanic participants in our data, due to limited opportunities to socially distance in multigenerational households and employment in public-facing roles during the pandemic (13). Likewise, individuals with lower household income and higher neighborhood deprivation also faced increased hospitalization risk, potentially due to fewer opportunities for remote work and barriers to physical distancing (14, 15). Moreover, there is evidence that US states with higher levels of income inequality experienced greater COVID-19 mortality, further indicating the role of structural inequity in pandemic outcomes via mechanisms such as healthcare quality and access (16).

Our finding that area deprivation was associated with COVID-19 hospitalization supports earlier analyses. Area-level variables including income, household size, population density (17, 18), low insurance coverage (18, 19), and higher air pollution levels (20, 21) have been found to be associated with COVID-19 morbidity and mortality, and our analysis contributes to this body of literature.

Our analysis found no significant association between education level and COVID-19 hospitalization. Previous studies have linked lower educational attainment with lower COVID-19 vaccine uptake (22) and overall health literacy (23, 24). However, there is evidence that the protective effect of education was greatest in the early stages of the pandemic (2020 to 2021), but that this effect waned as “information (on COVID-19) became more coherent and univocal, and/or people learned from their and their neighbors’ experience what strategies were more effective, so that education ended up being less relevant” (25). As our study only includes data from 2021 onward, an analysis of COVID-19 hospitalization data from 2020 is warranted to explore whether education conferred a protective effect against infection and hospitalization in this cohort in the early stages of the pandemic.

A key contribution of this study is the scale of its analysis across multiple social determinants of COVID-19 hospitalization within a single large, diverse U.S. cohort. Moreover, COVID-19 incidence and outcomes based on standardized EHR data rather than self-report allow for simultaneous consideration of individual and neighborhood-level socioeconomic factors. While prior studies often examined one or two socioeconomic factors or were restricted to a single region, this analysis evaluated income, education, and neighborhood deprivation simultaneously within the same model, enabling direct comparison of their independent effects. Moreover, while COVID-19 risk has been studied using *All of Us* data (26), our analysis is the first to consider the relationship between socioeconomic characteristics and risk of hospitalization in the cohort.

Our work has its limitations. It is likely that the true number of cases, hospitalizations, and COVID-19 vaccination uptake in this cohort is underrepresented due to incomplete EHR linkage. Our conservative definition of hospitalization, which was limited to primary inpatient encounters, may have excluded secondary COVID-19 admissions and thereby led to an underestimation of total hospitalizations. To capture both pre- and post-vaccine dynamics, we restricted the study period to 2021–2023; however, this approach may have excluded early hospitalization patterns from the initial phase of the pandemic in 2020 (27). Additionally, we did not stratify hospitalizations by COVID-19 variant, which may have influenced observed risk patterns across the study period. Finally, linkage to area deprivation data at a resolution greater than the 3-digit zip code prefix (currently the only geographic identifier in the cohort) would confer a more robust analysis.

In this nationwide sample, risk of COVID-19 hospitalization was closely associated with several demographic and socioeconomic factors. As COVID-19 remains a salient public health concern in the United States, policies and interventions may bolster their impact by focusing on older adults, racial/ethnic minorities, those with lower income, and those living in neighborhoods of greater deprivation.

## Data Availability

The original contributions presented in the study are included in the article/supplementary material, further inquiries can be directed to the corresponding author.
